# NCLcomparator: systematically post-screening non-co-linear transcripts (circular, *trans*-spliced, or fusion RNAs) identified from various detectors

**DOI:** 10.1186/s12859-018-2589-0

**Published:** 2019-01-03

**Authors:** Chia-Ying Chen, Trees-Juen Chuang

**Affiliations:** Genomics Research Center, Academia Sinica, Taipei, 11529 Taiwan

**Keywords:** RNA-seq, Non-co-linear RNA, Circular RNA, *Trans*-spliced RNA, Gene fusion, Alignment ambiguity

## Abstract

**Background:**

Non-co-linear (NCL) transcripts consist of exonic sequences that are topologically inconsistent with the reference genome in an intragenic fashion (circular or intragenic *trans*-spliced RNAs) or in an intergenic fashion (fusion or intergenic *trans*-spliced RNAs). On the basis of RNA-seq data, numerous NCL event detectors have been developed and detected thousands of NCL events in diverse species. However, there are great discrepancies in the identification results among detectors, indicating a considerable proportion of false positives in the detected NCL events. Although several helpful guidelines for evaluating the performance of NCL event detectors have been provided, a systematic guideline for measurement of NCL events identified by existing tools has not been available.

**Results:**

We develop a software, NCLcomparator, for systematically post-screening the intragenic or intergenic NCL events identified by various NCL detectors. NCLcomparator first examine whether the input NCL events are potentially false positives derived from ambiguous alignments (i.e., the NCL events have an alternative co-linear explanation or multiple matches against the reference genome). To evaluate the reliability of the identified NCL events, we define the NCL score (*NCL*_*score*_) based on the variation in the number of supporting NCL junction reads identified by the tools examined. Of the input NCL events, we show that the ambiguous alignment-derived events have relatively lower *NCL*_*score*_ values than the other events, indicating that an NCL event with a higher *NCL*_*score*_ has a higher level of reliability. To help selecting highly expressed NCL events, NCLcomparator also provides a series of useful measurements such as the expression levels of the detected NCL events and their corresponding host genes and the junction usage of the co-linear splice junctions at both NCL donor and acceptor sites.

**Conclusion:**

NCLcomparator provides useful guidelines, with the input of identified NCL events from various detectors and the corresponding paired-end RNA-seq data only, to help users selecting potentially high-confidence NCL events for further functional investigation. The software thus helps to facilitate future studies into NCL events, shedding light on the fundamental biology of this important but understudied class of transcripts. NCLcomparator is freely accessible at https://github.com/TreesLab/NCLcomparator.

**Electronic supplementary material:**

The online version of this article (10.1186/s12859-018-2589-0) contains supplementary material, which is available to authorized users.

## Background

Transcriptome-wide analyses of high-throughput RNA sequencing (RNA-seq) have discovered a large amount of ‘non-co-linear’ (NCL) transcripts, in which the exonic sequences are topologically inconsistent with the reference genome in an intragenic fashion (circular or intragenic *trans*-spliced RNAs) or in an intergenic fashion (fusion or intergenic *trans*-spliced RNAs) [1–4]. Although NCL transcripts were reported to be generally expressed at a rather low level compared with co-linear mRNAs, some NCL transcripts may be even more highly expressed than their corresponding co-linear isoforms [5] or evolutionarily conserved across species [6]. Accumulating evidence shows their biological importance in gene regulation and disease diagnosis [4, 7–9]. For fusion transcripts, some were demonstrated to correlate with malignant hematological disorders and sarcomas [10–13]. *BCR-ABL1*, a prominent example of fusion gene, was shown to be important in adult acute lymphoblastic leukemia cases and served as an effective biomarker for chronic myeloid leukemia [14–17]. For *trans*-spliced RNAs, some may play a role in anti-apoptotic function [3, 18, 19] and prostate cancer [3, 20]. A *trans*-spliced long non-coding RNA, tsRMST, can regulate pluripotency maintenance of human embryonic stem cells (hESCs) by repressing WNT5A [7, 21]. For circular RNAs (circRNAs), they are ubiquitous and have been observed in diverse species [5, 22–27]. The most famous function of circRNAs is their regulatory role in microRNA sponges [6, 28–32]. In addition, circRNAs can regulate their parent genes [4, 8, 33–35], or play a regulatory role in development [26, 36, 37], the aging nervous system [38], and cancer growth/metastasis [32, 39].

Nowadays, numerous RNA-seq-based NCL event detectors have been developed and employed to identify thousands of NCL transcript candidates in diverse species [40–50]. However, detection of NCL events is still hampered by the potentially false calls arising from sequencing errors, ambiguous alignment, and in vitro artifacts, which leads to great discrepancies in the detection results among tools [4, 51–53]. In addition, the biogenesis and functions of circRNAs and *trans*-spliced RNAs are mostly unclear. Even if the computationally identified NCL events are in vivo, it remains debatable whether most of them are merely side-products of imperfect pre-mRNA splicing [24, 54]. As accumulating NCL events are detected, the reliability and function of the identified NCL events become an unavoidable question for further investigation. Although several studies have provided helpful guidelines for evaluating the performance of various NCL event-detection tools [1, 4, 51, 55, 56], a systematic guideline for measurement of NCL events identified by different tools has not been available. To reduce the cost of further validation and functional analysis, it is essential to systematically evaluate the reliability of the detected NCL events.

To provide useful guidelines on screening the NCL events identified by various detectors for users, we develop an analysis package, NCLcomparator, for systematic comparisons of the outputs from different detectors. First, for each input NCL event, NCLcomparator concatenates the sequence flanking the NCL junction and then examines whether this NCL event is potentially false positives derived from ambiguous alignments by aligning the concatenated sequence against the reference genome. Next, on the basis of the number of the supporting NCL junction reads derived from the tools compared, NCLcomparator defines the NCL score, *NCL*_*score*_, to evaluate the reliability of the input NCL events. To help selecting highly expressed NCL events, NCLcomparator provides expression levels of NCL events and their corresponding co-linear host genes and calculates the ratio of the number of reads spanning the NCL junction to that spanning the co-linear splice junctions at both NCL donor and acceptor sites. NCLcomparator further estimates the frequencies of occurrence of the co-linear junctions at the NCL donor and acceptor splicing sites in the host genes to examine the usage of the NCL junctions. NCLcomparator also provides the number of the mapped paired-end read with a read spanning outside the identified intragenic circle, which can be regarded as a good indicator for discrimination between circRNAs and intragenic *trans*-spliced RNAs [4]. Taken together, NCLcomparator is helpful not only for selecting highly confident and highly expressed NCL events but also for further investigating biogenesis and function of this important but understudied class of transcripts. Of note, NCLcomparator analyzes both intragenic and intergenic NCL events, allowing researchers for comparisons among circRNA detectors and among gene-fusion detectors.

## Implementation

**Fig. 1:**
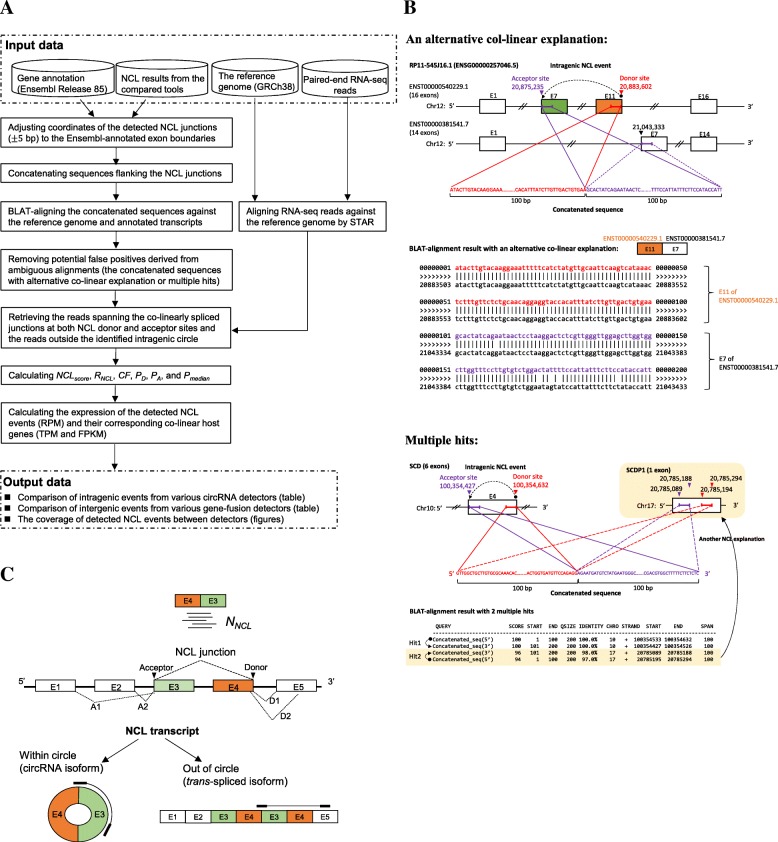
The NCLcomparator process. **a** Flowchart of NCLcomparator. **b** Examples of NCL events derived from ambiguous alignments with an alternative co-linear explanation (top) and multiple hits (bottom). For the top panel, the host gene (RP11-545 J16.1) includes two alternatively spliced transcripts (ENST00000540229.1 with 16 exons and ENST00000381541.7 with 14 exons). The concatenated sequence of the intragenic NCL event (E11-E7; ENST00000540229.1) has an alternative co-linear explanation (E11 of ENST00000540229.1 and E7 of ENST00000381541.7). For the bottom panel, the concatenated sequence of the intragenic NCL event (E4-E4; SCD) mapped to multiple positions (2 hits). **c** Schematic illustration of the NCL junction and the corresponding co-linear junctions at both NCL donor (D1 and D2) and acceptor (A1 and A2) sites. The mapped read spanning within (circRNA isoform) or outside (*trans*-spliced isoform) a detected intragenic circle is illustrated. E, exon. *N*_*NCL*_, number of reads spanning the NCL junction

The flowchart of the NCLcomparator pipeline is listed in Fig. 1a. The input data include the identified NCL events from various detectors and the corresponding paired-end RNA-seq data. The input data for each tool should include the coordinates of the detected NCL donor/acceptor sites and the number of reads spanning the NCL junction (*N*_*NCL*_). NCLcomparator only considers the detected NCL events in which splice junctions agree to well-annotated junctions (co-linear) for comparisons for two reasons. First, such events were reported to be more reliable [2, 7, 40, 49] and second, some tools only detect NCL donor/acceptor sites at known co-linear exon boundaries (e.g., NCLscan [1] and UROBORUS [57]). To reduce possible alignment errors around the splice junctions among tools, an NCL event is recorded when the distance between the known co-linear junction and junction identified by the tested tool is equal to or less than 5 bp, in which the coordinates of the NCL junction is adjusted to those of the well-annotated boundary.

Since repetitive sequences or paralogous genes often masquerade as NCL events due to ambiguous alignments of short RNA-seq reads [1, 25, 58–60], NCLcomparator checks the alignment ambiguity of the input NCL events and removes such potentially false positives. To this end, for each input NCL event, NCLcomparator concatenates the exonic sequence flanking the NCL junction (within − 100 nucleotides to + 100 nucleotides of each NCL junction) and then aligns the 200 bp concatenated sequence against the reference genome and well-annotated transcripts using BLAT [61]. Of note, the concatenated sequence may be shorter than 200 bp if the flanking exonic circRNA sequence is shorter than 200 bp. A concatenated sequence is regarded as false positives derived from ambiguous alignments, if it contains at least an alternative co-linear explanation (the sequence similarity of the alternative co-linear explanation is more than 80% identical to that of the non-co-linear one; Fig. 1b, top) or maps to multiple positions with similar BLAT mapping scores (difference of BLAT-mapping scores < 3; Fig. 1b, bottom).

To extract the reads spanning the co-linearly spliced junctions at both NCL donor and acceptor sites (Fig. 1c) according to the adjusted NCL junctions, the paired-end RNA-seq reads are aligned against the reference genome using STAR with the ‘chimeric alignment’ model [62]. The reads mapping outside the identified intragenic circle are also extracted, which is often employed to distinguish between *trans*-splicing and circRNA events [4] (Fig. 1c). In addition, to evaluate the variation in *N*_*NCL*_ identified by the tools compared, we define *τ*_*NCL*_ as.

1 $$ \frac{\sum \limits_{i=1}^n\left(1-\left(\frac{\log \left({N}_{NCL}(i)+1\right)}{\log \left(\operatorname{Max}\left({N}_{NCL}\right)+1\right)}\right)\right)}{n-1} $$

where *n* is the number of tools compared, *N*_*NCL*_(*i*) indicates *N*_*NCL*_ of the NCL event of interest identified by Tool *i*, and Max(*N*_*NCL*_) is the highest *N*_*NCL*_ of the NCL event across all examined tools. Of note, *τ*_*NCL*_ of an NCL event is defined as the heterogeneity of its *N*_*NCL*_ value provided by the tools compared, which ranges from 0 to 1 with higher *τ*_*NCL*_ values indicating greater variation (or higher tool-specificity) in *N*_*NCL*_. The measurement of *τ*_*NCL*_ value is similar to that applied for evaluating sample specificity of DNA methylation level [63]. The NCL score, *NCL*_*score*_, is then defined as.

2 $$ {\log}_{10}\frac{Median{\left({N}_{NCL}\right)}^2+\kappa }{\tau_{NCL}+\kappa } $$

where Median(*N*_*NCL*_) is the median *N*_*NCL*_ of an NCL event across all examined tools and κ is a pseudocount arbitrarily set as 0.01 to avoid the occurrence of undefined values. A higher *NCL*_*score*_ of an NCL event indicates a greater median *N*_*NCL*_ with a smaller variation (*τ*_*NCL*_) in *N*_*NCL*_ among tools compared, suggesting a higher level of confidence. To quantify the abundance of each detected NCL event as compared with that of its corresponding co-linear isoform(s), we calculate NCL ratio (*R*_*NCL*_) [59] and circular fraction (CF) [24] using *N*_*NCL*_ and the number of reads spanning the co-linearly spliced junctions at both NCL donor (*N*_*D*_) and acceptor (*N*_*A*_) sites. *R*_*NCL*_ and CF are defined as

3 $$ \frac{2{N}_{NCL}}{2{N}_{NCL}+{N}_D+{N}_A}\ \mathrm{and}\ \frac{N_{NCL}}{N_{NCL}+{N}_D+{N}_A+1} $$

respectively. Noteworthily, both *R*_*NCL*_ and CF range from 0 to 1, with *R*_*NCL*_ > 0.5 or CF > ~ 1/3 indicating a higher expression level in a NCL isoform than in its corresponding co-linear isoform. To quantify the usage of the co-linear junctions at both NCL donor and acceptor splice sites in the corresponding host gene, we also define *P*_*D*_ and *P*_*A*_ as

4 $$ \frac{N_D}{\mathrm{all}\ \mathrm{co}\hbox{-} \mathrm{linear}\ \mathrm{junction}\ \mathrm{reads}}\ \mathrm{and}\ \frac{N_A}{\mathrm{all}\ \mathrm{co}\hbox{-} \mathrm{linear}\ \mathrm{junction}\ \mathrm{reads}} $$

respectively. “all co-linear junction reads” means the sum of reads spanning the co-linearly spliced junctions at all well-annotated splice sites in the host gene. For comparison, the median frequency (*P*_*median*_) of occurrence of all well-annotated splice sites (co-linear) in the host gene is also provided. The expression levels of NCL events are determined as the number of supporting reads per million raw reads (*RPM*_*raw*_) or per million uniquely mapped reads (*RPM*_*mapped*_) [64]; those of the corresponding co-linear host gene are estimated by transcripts per million (TPM) and fragments per kilobase of transcript per million mapped reads (FPKM) using RSEM [65]. Since synonymous constraint elements (SCEs) were suggested to be important in RNA secondary structures, RNA splicing, microRNA binding, and nucleosome positioning [66, 67], we also determine whether the NCL donor and acceptor junctions are located within SCEs. The union of the detected NCL events from the compared tools is exported into two tab-delimited text files (intragenic and intergenic results, respectively), in which the related information stated above is included. The figures representing coverage of identified NCL events among the compared tools and distribution of the number of supporting tools are also provided.

## Results

**Fig. 2:**
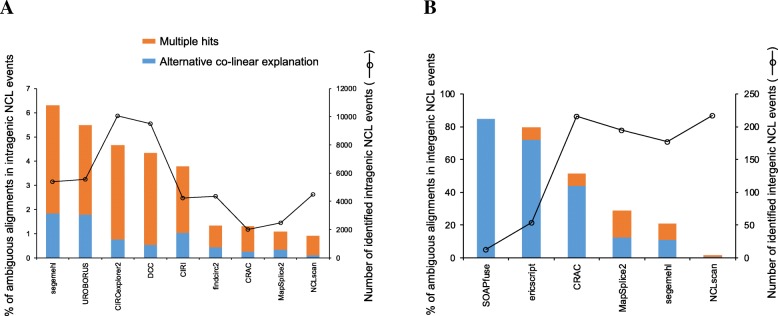
Distributions of (**a**) intragenic and (**b**) intergenic NCL events derived from ambiguous alignments with an alternative co-linear explanation and multiple hit(s) among various tools on the rRNA depleted RNA-seq data of HeLa cells (SRR1637089)

NCLcomparator is applied to an rRNA depleted RNA-seq of HeLa cells (SRR1637089). Nine intragenic and six intergenic NCL detectors are selected and run independently with default parameters or the parameters suggested by the authors (Additional file 1: Table S1). Some tools can simultaneously detect intragenic and intergenic NCL events (e.g., NCLscan, Segemehl [68], and MapSplice [69]). These tools totally identified 17,313 intragenic and 766 intergenic NCL events (i.e., the input NCL events; Additional file 2: Table S2). NCLcomparator first checks the alignment ambiguity of the input NCL events. Of the 17,313 intragenic NCL events, 269 events contain alternative co-linear explanations and 792 events map to multiple positions with similar BLAT mapping scores (Table 1). Of the 766 intergenic NCL events, 184 and 69 events have alternative co-linear explanations and multiple hits, respectively (Table 1). We can find that the proportions of false positives derived from ambiguous alignments vary among the compared tools and are generally higher in intergenic NCL events than in intragenic NCL events (Fig. 2a and b), reflecting previous reports that many intergenic NCL events may arise from false positives (sequencing/alignment errors or in vitro artifacts) [7, 42]. Particularly, more than 50% of intergenic events identified by CRAC, ericscript, and SOAPfuse are derived from ambiguous alignments. Of note, the NCLscan results have the lowest percentages of false positives derived from ambiguous alignments among the results of the compared tools; such percentages are consistently low (~ 1%) in both intragenic and intergenic NCL event detections (Fig. 2a and b). This result consists with previous reports that NCLscan has the highest precision compared with other tools [1, 51]. The NCL events that are determined to be derived from ambiguous alignments (designated as “ambiguous NCL events”) are removed. Therefore, a total of 16,252 intragenic and 513 intergenic events (designated as “non-ambiguous NCL events”) are retained for the following comparisons (Table 1).

**Table 1:** The number of intragenic and intergenic NCL events before and after screening

	Number of NCL events
Intragenic NCL events	
Before screening	17,313
Alignment ambiguity (ambiguous NCL events)	1061
Alternative co-linear explanation	269
Multiple hit	792
After screening (non-ambiguous NCL events)	16,252
Intergenic NCL events	
Before screening	766
Alignment ambiguity (ambiguous NCL events)	253
Alternative co-linear explanation	184
Multiple hit	69
After screening (non-ambiguous NCL events)	513

**Fig. 3:**
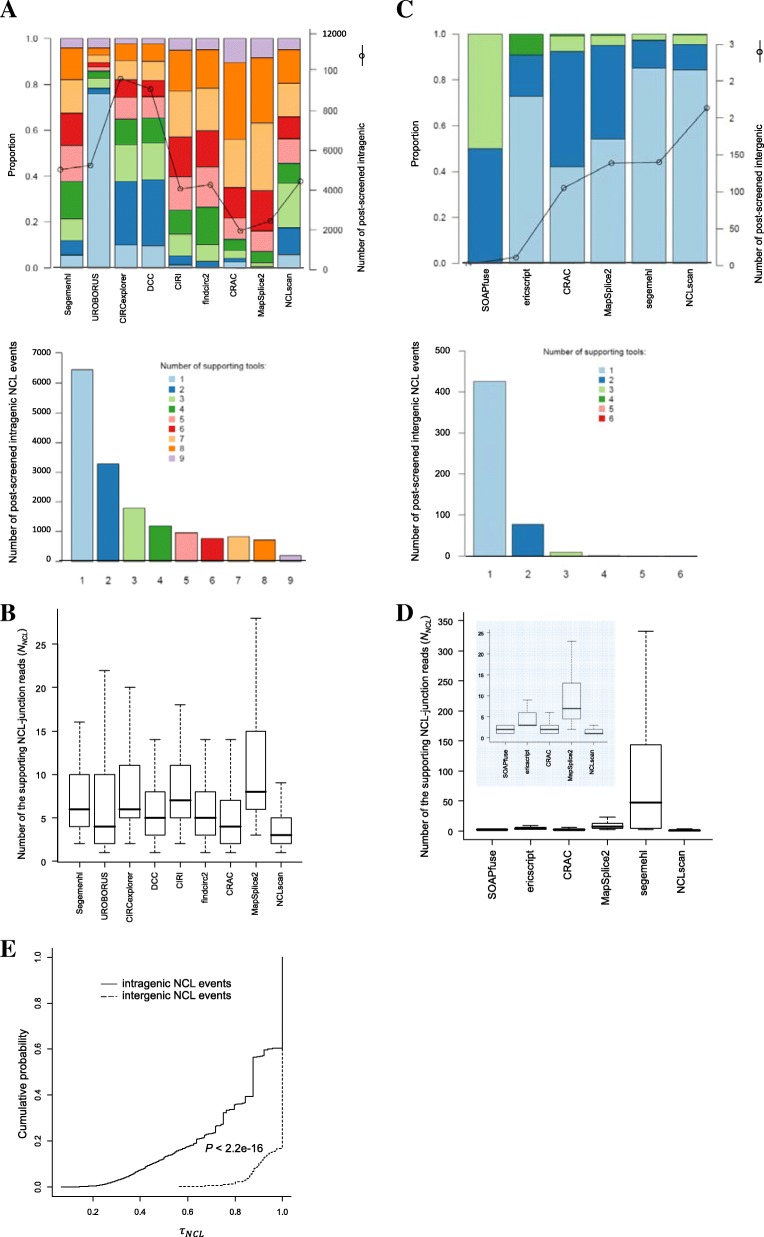
Variation in number of detected events and *N*_*NCL*_ among various tools. Of note, the analysis is based on the non-ambiguous NCL events. **a** The coverage of identified intragenic NCL events between the compared tools (top) and the distribution of the number of supporting tools (bottom). **b** Boxplot representing the number of the supporting NCL-junction reads (*N*_*NCL*_) of the intragenic NCL events (205 events) that are identified by all nine examined circRNA detectors. **c** and **d** representing the results of intergenic NCL events identified by 6 gene-fusion detectors, related to the intragenic cases in (**a**) and (**b**), respectively. For (**d**), boxplot represents the 87 intergenic NCL events identified by at least two gene-fusion detectors. A zoom-in view for *N*_*NCL*_ of SOAPfuse, ericscript, CRAC, MapSplice2, and NCLscan is shown the middle panel of (**d**). The identified intragenic and intergenic NCL events by various tools are listed in Additional file 2: Table S2. **e** Comparisons of cumulative distribution of *τ*_*NCL*_ for the non-ambiguous intragenic and intergenic NCL events. *P* value is determined by the Kolmogorov-Smirnov test

In addition to tab-delimited text files (e.g., Additional file 2: Table S2), NCLcomparator provides figures for comparison of identification results from different tools (see Fig. 3a and b for intragenic events and Fig. 3c and d for intergenic events). Here we take the result of intragenic NCL events as an example. We can find quite a large variation in numbers of detected events among tools; every tool identifies a considerable proportion of tool-specific intragenic NCL events (Fig. 3a, top). More than 40% (6450 events) of the intragenic NCL events are exclusively identified by a single tool (Fig. 3a, bottom). Particularly, even though the intragenic NCL events (205 events) detected by all the nine tools compared, the number of supporting NCL-junction reads (*N*_*NCL*_) vary among the compared tools (Fig. 3b). These observations reflect great discrepancies in the detection results (including the number and *N*_*NCL*_ values of identified events) among tools. Such great discrepancies are much more remarkable in intergenic events than in intragenic ones. We can find that except for SOAPfuse and ericscript every tool identifies more than 30% of tool-specific intragenic NCL events (Fig. 3c, top), more than 83% (426 out of 513) of intergenic events are exclusively identified by a single tool (Fig. 3c, bottom), and the *N*_*NCL*_ values highly vary among the compared tools (Fig. 3d). In addition, comparisons of the cumulative distribution of *τ*_*NCL*_show that *τ*_*NCL*_ values are significantly higher in intergenic events than in intragenic ones (*P* value < 2.2e-16 by the Kolmogorov-Smirnov test; Fig. 3e). These observations highlight the importance of a careful screen for the NCL events, especially for intergenic events, identified by currently available NCL event detectors.

**Fig. 4:**
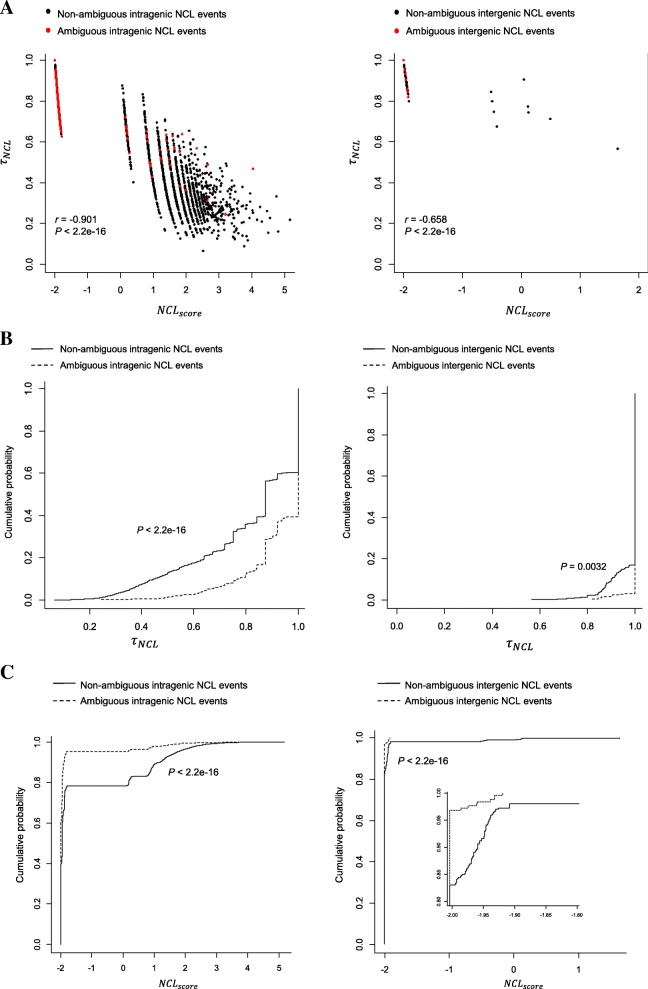
Comparisons of *τ*_*NCL*_ and *NCL*_*score*_ between ambiguous and non-ambiguous NCL events. **a** Correlation between *τ*_*NCL*_ and *NCL*_*score*_ for intragenic (left) and intergenic (right) NCL events. The black and red dots represent non-ambiguous and ambiguous NCL events, respectively. The correlation coefficient *r* and *P* values are determined by Pearson’s *r* test. **b** and **c** Comparisons of cumulative distribution of (**b**) *τ*_*NCL*_ and (**c**) *NCL*_*score*_ for the ambiguous and non-ambiguous NCL events in intragenic (left) and intergenic (right) detections. In (**c**), a zoom-in view is shown in the lower right panel. *P* values are determined by the Kolmogorov-Smirnov test

Importantly, NCLcomparator provides two measurements, *τ*_*NCL*_ and *NCL*_*score*_ (see Eqs. (1) and (2), respectively) to help users selecting potentially high- plausible NCL events. Since *N*_*NCL*_ values vary remarkably between tools depending on the level of strictness of the filtering steps used [51, 60] (see also Fig. 3b and d), we speculate that high-confidence NCL events tend to have a large *NCL*_*score*_ (in other words, a large median *N*_*NCL*_ with a small *τ*_*NCL*_). Since ambiguous NCL events can be regarded as false positives, we examine *τ*_*NCL*_and *NCL*_*score*_ values of ambiguous and non-ambiguous NCL events. Indeed, we find a significantly negative correlation between these two measurements, in which *τ*_*NCL*_ values are significantly higher in ambiguous NCL events than in non-ambiguous ones (Fig. 4a and b), whereas the reverse trends are observed for *NCL*_*score*_ values (Fig. 4a and c), regardless of whether the events are intragenic or intergenic (both *P* values < 2.2e-16 by the Kolmogorov-Smirnov test). For example, for *τ*_*NCL*_*τ*_*NCL*_, more than 95% of ambiguous events are filtered out, if we set the thresholds as < 0.6 and < 1 for intragenic and intergenic events, respectively (Fig. 4b). Meanwhile, for *NCL*_*score*_, more than 95% of ambiguous events are removed, if we set the thresholds as > − 1 and > − 2 for intragenic and intergenic events, respectively (Fig. 4c). These results reveal that *NCL*_*score*_ is a good indicator for selecting NCL events with high level of confidence, suggesting that NCL events with a large *NCL*_*score*_ may be of high reliability and considered to take priority over the other for further experimental validation and functional analysis.

## Discussion

There are several major challenges for detection of NCL events. In addition to false positives arising from alignment ambiguity and biased identification of NCL events from different bioinformatics approaches as stated above, identification of NCL events is often hampered by in vitro artifacts, particularly template switching during reverse transcription (RT) [2, 7, 42, 45, 59, 70, 71]. Actually, to minimize potential RT-artifacts, it would be better to confirm identified NCL events using both RT- and non-RT-based experiments (e.g., Northern blot or RNase protection assay [72]). However, it is required to develop a method for systematic identification of NCL events with controlling for experimental artifacts. While a study successfully detected a huge number of experimental artifacts based on *Drosophila* hybrid mRNAs (*D. melanogaster* females vs. *D. sechellia* males) and a mixed mRNA-negative control sample [42], this approach would not be applied to human studies. Alternatively, it has been demonstrated that RTase-dependent RNA products are likely to be RT artifacts [2, 4, 7, 73, 74]. RT-based artifacts can be detected by comparisons of different RTase products, which was shown to serve as effectively as RTase-free validation [2, 7]. On the basis of comparisons of Avian Myeloblastosis Virus- and Moloney Murine Leukemia Virus-derived RTase products, a recent study successfully applied this concept to human samples and systematically identified NCL events with controlling for experimental artifacts [59].

Moreover, NCL junctions can be generated during post-transcriptional processes (*trans*-spliced or circular RNAs) or by genetic rearrangements (fusion RNAs) at the DNA level. Thus, discrimination between post-transcriptional NCL events and genetic rearrangements presents another challenge to detection/analysis of NCL events. Since NCL events that are observed in multiple biological samples or conserved across multiple species are less likely to be formed by somatic recombination, post-transcriptional NCL events may be extracted by this simple rule [2, 7]. A more efficient approach is to analyze both RNA-seq data and whole genome sequencing (WGS) data from the same sample. Some systematic pipelines have been developed, which integrated WGS-based rearrangement detection with RNA-seq-based NCL detection to identify fusion RNAs, and successfully applied to analysis of functionally recurrent gene fusions in human diseases [75–80]. While many studies have focused on identification/analysis of fusion RNAs that consist of sequence fragments from different genes, transcribed rearrangements in an intragenic fashion is relatively less investigated.

With more and more NCL events are identified, the reliability and function of such a large number of NCL events remains an open question worthy of further investigation. To reduce the cost of subsequent validation and functional analysis, carefully evaluating the reliability of detected NCL events with considering all abovementioned challenges awaits further development.

## Conclusion

Dozens of RNA-seq-based detectors have been developed and successfully identify thousands of NCL transcript candidates (circular, *trans*-spliced, or fusion RNA) in diverse species. However, there are great discrepancies in the identification results (including the number of NCL events and the number of the supporting NCL junction reads of the identified events) among tools, indicating a considerable proportion of potentially false positives in the results. NCLcomparator screens out potentially false positives originating from ambiguous alignments and provides a series of useful measurements, including NCL score (*NCL*_*score*_), NCL ratio (*R*_*NCL*_), circular fraction (CF), the usage of the co-linear junctions at both NCL donor and acceptor splice sites in the corresponding host gene (*P*_*D*_, *P*_*A*_, and *P*_*median*_), and the expression levels of NCL events (*RPM*_*raw*_ and *RPM*_*mapped*_) and their corresponding co-linear host genes (FPKM and TPM), for users to screen the NCL events from various detectors. On the basis of the NCLcomparator-provided information, users can easily select potentially high-plausible NCL candidates with a high expression level and/or a low variation of supporting NCL junction reads from multiple NCL detectors. The software, a post-processing tool for screening identified NCL events from existing detectors, thus help to facilitate future studies into NCL events, shedding light on the fundamental biology of this important but understudied class of transcripts.

## Availability and requirements

Project name: NCLcomparator.

Project home page: https://github.com/TreesLab/NCLcomparator

Operator system(s): Linux-like environment (Bio-Linux).

Programming language: shell script.

Other requirement: None.

License: None.

Any restrictions to use by non-academics: None.

Data: The tested RNA-seq data was derived from HeLa cells with rRNA depletion, which was downloaded from the NCBI Sequence Read Archive (SRR1637089) at https://trace.ddbj.nig.ac.jp/DRASearch/run?acc=SRR1637089. All parameter settings and identification results of intragenic/intergenic NCL detectors tested in this study are reported in Additional file 1: Table S1 and Additional file 2: Table S2, respectively.

## Additional files

Additional file 1: **Table S1.** Parameter settings of intragenic/intergenic NCL detectors tested in this study. (DOCX 34 kb)

Additional file 2: **Table S2.** The totally identified 17,313 intragenic and 766 intergenic NCL events by the 9 intragenic and 6 intergenic NCL detectors on the RNA-seq data of HeLa cells (XLSX 6083 kb)

## Abbreviations

circRNA: Circular RNA
FPKM: Fragments per kilobase of transcript per million mapped reads
NCL: Non-co-linear
RNA-seq: RNA sequencing
SCEs: Synonymous constraint elements
TPM: Transcripts per million
WGS: Whole genome sequencing
