# Supplementary material for: NCLcomparator: systematically post-screening non-co-linear transcripts (circular, trans-spliced, or fusion RNAs) identified from various detectors
Source: BMC Bioinformatics. 2019 Jan 3;20:3. doi: 10.1186/s12859-018-2589-0 (PMC6318855; doi:10.1186/s12859-018-2589-0)
Supplement: Supplementary file 1 — Table S1. Parameter settings of intragenic/intergenic NCL detectors tested in this study. (DOCX 34 kb) [file 12859_2018_2589_MOESM1_ESM.docx]

**Table S1.** Parameters of intragenic/intergenic NCL detectors examined in this study.

| **Method/version**  **[reference]** | **Intragenic/intergenic** | **Command line** |
| --- | --- | --- |
| NCLscan v2.0 [1] | Intragenic/intergenic | $ ./NCLscan.py -c /path/to/NCLscan.config --fq1 HeLa_1.fastq --fq2 HeLa_2.fastq -pj HeLa -o HeLa |
| Segemehl v0.2.0 [2] | Intragenic/intergenic | $ mkdir HeLa  $ ./segemehl.x - S - O -i hg38.idx -d hg38.fa -q HeLa_1.fastq -p HeLa_2.fastq -t 20 -o HeLa/HeLa.sam  $ ./testrealign.x -d hg38.fa -q HeLa/HeLa.sam –n  $ python filterjunction.py -u splicesties.bed -t transrealigned.bed -M -F -m 2 -T > HeLa/HeLa_transspliced_filtered.bed  $ python filterjunctions.py -u splicesties.bed -t transrealigned.bed -M -F -m 2 -C > HeLa/HeLa_circular_filtered.bed |
| MapSplice v2.2.0 [3] | Intragenic/intergenic | $ python mapsplice.py --qual-scale phred33 --fusion --min-fusion-distance 200 --gene-gtf /path/to/Homo_sapiens.GRCh38.87.gtf -c /path/to/hg38_chroms -x /path/to/bowtieIndex -1 HeLa_1.fastq -2 HeLa_2.fastq -p 20 -o HeLa |
| CRAC [9] | Intragenic/intergenic | $ cat HeLa_1.fastq HeLa_2.fastq > HeLa.fastq  $ mkdir HeLa  $ /path/to/crac -i hg38 -k 22 -r HeLa.fastq -o HeLa/HeLa.sam --nb-threads 20 –chimera HeLa/HeLa.chimera --stringent-chimera |
| CIRI v2.0.2 [4] | Intragenic | $ bwa mem /path/to/hg38.fa /path/to/HeLa_1.fastq /path/to/HeLa_2.fastq -T 10 1> HeLa_aln-pe.sam 2> HeLa_aln-pe.log  $ perl /path/to/CIRI_v2.0.2.pl -I HeLa_aln-pe.sam -O HeLa -F /path/to/hg38.fa -A /path/to/Homo_sapiens.GRCh38.87.gtf  rm -r -f HeLa_aln-pe.sam |
| CIRCexplorer2 v2.2.3 [5] | Intragenic | $ tophat2 -o HeLa -a 6 --microexon-search -m 2 -p 10 -G /path/to/Homo_sapiens.GRCh38.87.gtf /path/to/ bowtie2_index/hg38 /path/to/HeLa_1.fastq /path/to/HeLa_2.fastq  $ bamToFastq -i HeLa/unmapped.bam -fq HeLa/unmapped.fastq  $ tophat2 -o HeLa_fusion -p 10 --fusion-search --keep-fasta-order --bowtie1 --no-coverage-search /path/to/bowtie1_index/hg38 HeLa/unmapped.fastq  $ CIRCexplorer2 parse -t TopHat-Fusion HeLa_fusion/accepted_hits.bam > CIRCexplorer2_parse_HeLa.log  $ CIRCexplorer2 annotate -r hg38_ref.txt -g hg38.fa circ_out > CIRCexplorer2_annotate_HeLa.log |
| find_circ v2 [6] | Intragenic | $ mkdir HeLa  $ cd HeLa  $ bowtie2 -p10 --very-sensitive --mm -M20 --score-min=C,-15,0 -x /path/to/bowtie2_index/hg38 -1 /path/to/HeLa_1.fastq -2 /path/to/HeLa_2.fastq 2> bt2_firstpass.log \| samtools view -hbuS - \| samtools sort - HeLa  samtools view -hf 4 HeLa.bam \| samtools view -Sb - > unmapped_HeLa.bam  $./unmapped2anchors.py unmapped_HeLa.bam > HeLa_anchors.qfa  $ bowtie2 -p 10 --score-min=C,-15,0 --reorder --mm -q –U HeLa_anchors.qfa -x /path/to/bowtie2_index/hg38 \|./find_circ.py genome=/path/to/hg38.fa --prefix=HeLa_ --name=HeLa_sample --stats=HeLa/stats.txt --reads=HeLa/spliced_reads.fa > HeLa/splice_sites.bed  $ grep CIRCULAR HeLa/splice_sites.bed \| grep -v chrM \| awk '$5>=2' \| grep UNAMBIGUOUS_BP \| grep ANCHOR_UNIQUE \| ./maxlength.py 100000 > HeLa/circ_candidates.bed |
| DCC [7] | Intragenic | $ mkdir HeLa  $ cd HeLa  $ STAR --runThreadN 6 --genomeDir /path/to/star_index_hg38/ --outSAMtype BAM Unsorted --readFilesIn HeLa_1.fastq HeLa_2.fastq --outFileNamePrefix HeLa --outReadsUnmapped Fastx --outSJfilterOverhangMin 15 15 15 15 --alignSJoverhangMin 15 --alignSJDBoverhangMin 15 --outFilterMultimapNmax 20 --outFilterScoreMin 1 --outFilterMatchNmin 1 --outFilterMismatchNmax 2 --chimSegmentMin 15 --chimScoreMin 15 --chimScoreSeparation 10 --chimJunctionOverhangMin 15  $ cd ..  $ mkdir HeLa_mate1  $ cd HeLa_mate1  $ STAR --runThreadN 6 --genomeDir /path/to/star_index_hg38/ --outSAMtype BAM Unsorted --readFilesIn HeLa_1.fastq --outFileNamePrefix HeLa_mate1 --outReadsUnmapped Fastx --outSJfilterOverhangMin 15 15 15 15 --alignSJoverhangMin 15 --alignSJDBoverhangMin 15 --seedSearchStartLmax 30 --outFilterMultimapNmax 20 --outFilterScoreMin 1 --outFilterMatchNmin 1 --outFilterMismatchNmax 2 --chimSegmentMin 15 --chimScoreMin 15 --chimScoreSeparation 10 --chimJunctionOverhangMin 15  $ cd ..  $ mkdir HeLa_mate2  $ cd HeLa_mate2  $ STAR --runThreadN 6 --genomeDir /path/to/star_index_hg38/ --outSAMtype BAM Unsorted --readFilesIn HeLa_2.fastq --outFileNamePrefix HeLa_mate2 --outReadsUnmapped Fastx --outSJfilterOverhangMin 15 15 15 15 --alignSJoverhangMin 15 --alignSJDBoverhangMin 15 --seedSearchStartLmax 30 --outFilterMultimapNmax 20 --outFilterScoreMin 1 --outFilterMatchNmin 1 --outFilterMismatchNmax 2 --chimSegmentMin 15 --chimScoreMin 15 --chimScoreSeparation 10 --chimJunctionOverhangMin 15  $ cd ..  $ mkdir HeLa_DCC  cd HeLa_DCC  $ DCC /path/to/HeLa/HeLaChimeric.out.junction -mt1 /path/to/HeLa_mate1/HeLa_mate1Chimeric.out.junction -mt2 /path/to/HeLa_mate2/HeLa_mate2Chimeric.out.junction -D -R /path/to/hg38_Repeats.gtf –an /path/to/gencode.v24.annotation.gtf -Pi -F -M -Nr 1 1 -fg -G -A /path/to/hg38.fa |
| UROBORUS [8] | Intragenic | $ tophat -p 6 --bowtie1 -o HeLa_tophat /path/to/bowtie1_index_hg38/hg38 HeLa_1.fastq HeLa_2.fastq  $ samtools view -o HeLa_tophat/unmapped.sam HeLa_tophat/unmapped.bam  $ perl /path/to/UROBORUS.pl -index /path/to/bowtie1_index_hg38/hg38 –gtf gencode_GRCh38.v24.gtf -fasta /path/to/bowtie1_index_hg38 HeLa_tophat/unmapped.sam |
| SOAPfuse v1.27 [10] | Intergenic | $ perl SOAPfuse-RUN.pl -c /path/to/SOAPfuse-v1.27/config/config.txt -fd /path/to/SOAPfuse-v1.27/fastq_data -l sample.list -o RunOut |
| Ericscript v0.5.5 [11] | Intergenic | $ ericscript.pl -db /path/to/ericscript_db_homosapiens_ensembl84' -name GM12878 -o RunOut /path/to/GM12878_cell_nonPolyA_1.fastq /path/to/GM12878_2.fastq |

**References**

1. Chuang TJ, Wu CS, Chen CY, Hung LY, Chiang TW, Yang MY: **NCLscan: accurate identification of non-co-linear transcripts (fusion, trans-splicing and circular RNA) with a good balance between sensitivity and precision**. *Nucleic acids research* 2016, **44**(3):e29.

2. Hoffmann S, Otto C, Doose G, Tanzer A, Langenberger D, Christ S, Kunz M, Holdt LM, Teupser D, Hackermuller J *et al*: **A multi-split mapping algorithm for circular RNA, splicing, trans-splicing and fusion detection**. *Genome biology* 2014, **15**(2):R34.

3. Wang K, Singh D, Zeng Z, Coleman SJ, Huang Y, Savich GL, He X, Mieczkowski P, Grimm SA, Perou CM *et al*: **MapSplice: accurate mapping of RNA-seq reads for splice junction discovery**. *Nucleic acids research* 2010, **38**(18):e178.

4. Gao Y, Wang J, Zhao F: **CIRI: an efficient and unbiased algorithm for de novo circular RNA identification**. *Genome Biol* 2015, **16**:4.

5. Zhang XO, Wang HB, Zhang Y, Lu X, Chen LL, Yang L: **Complementary sequence-mediated exon circularization**. *Cell* 2014, **159**(1):134-147.

6. Memczak S, Jens M, Elefsinioti A, Torti F, Krueger J, Rybak A, Maier L, Mackowiak SD, Gregersen LH, Munschauer M *et al*: **Circular RNAs are a large class of animal RNAs with regulatory potency**. *Nature* 2013, **495**(7441):333-338.

7. Cheng J, Metge F, Dieterich C: **Specific identification and quantification of circular RNAs from sequencing data**. *Bioinformatics (Oxford, England)* 2016, **32**(7):1094-1096.

8. Song X, Zhang N, Han P, Moon BS, Lai RK, Wang K, Lu W: **Circular RNA profile in gliomas revealed by identification tool UROBORUS**. *Nucleic acids research* 2016, **44**(9):e87.

9. Philippe N, Salson M, Commes T, Rivals E: **CRAC: an integrated approach to the analysis of RNA-seq reads**. *Genome biology* 2013, **14**(3):R30.

10. Jia W, Qiu K, He M, Song P, Zhou Q, Zhou F, Yu Y, Zhu D, Nickerson ML, Wan S *et al*: **SOAPfuse: an algorithm for identifying fusion transcripts from paired-end RNA-Seq data**. *Genome biology* 2013, **14**(2):R12.

11. Benelli M, Pescucci C, Marseglia G, Severgnini M, Torricelli F, Magi A: **Discovering chimeric transcripts in paired-end RNA-seq data by using EricScript**. *Bioinformatics* 2012, **28**(24):3232-3239.
